# Characteristics of Sudden Cardiac Arrest in Young Athletes: A Web-Based Survey of Athletes in Japanese College Sports

**DOI:** 10.1155/crp/1265728

**Published:** 2025-10-10

**Authors:** Shuhei Yano, Yoshinori Katsumata, Yuki Muramoto, Akira Kinoda, Takeshi Kimura, Kazuki Sato, Masaki Ieda

**Affiliations:** ^1^Department of Cardiology, Keio University School of Medicine, Tokyo, Japan; ^2^Institute for Integrated Sports Medicine, Keio University School of Medicine, Tokyo, Japan

## Abstract

**Background:**

Sudden death in young athletes is a serious concern, and appropriate, noninvasive, and easily implementable screening of at-risk individuals is imperative.

**Objectives:**

This study aimed to identify potential risk factors associated with sudden cardiac arrest (SCA) in collegiate athletes.

**Methods:**

In this cross-sectional observational study, we conducted an online survey of college student athletes who were part of Japanese university sports organizations associated with the Japanese Collegiate Athletic Association (UNIVAS) between June 2022 and October 2022. The questionnaire collected information on prior cardiac arrest and personal and family medical history.

**Results:**

A total of 10,861 athletes (median age: 19.9 years; female: 37.2%) answered the questionnaire. Six athletes (three males and three females) reported a history of cardiac arrest. Of the six patients, two had a history of arrhythmia and four had a history of syncope. Arrhythmia and syncope were significantly more common in athletes with SCA (*p* < 0.01). Similarly, a family history of heart failure, arrhythmia, or syncope was significantly more common in patients with SCA (*p* < 0.01), and a history of previous syncope significantly increased the odds ratio for the occurrence of SCA (odds ratio: 41.98; 95% confidence interval: 5.99–293.83, *p* < 0.01).

**Conclusions:**

A history of syncope significantly increases the risk of SCA in young athletes. Further research is needed to stratify the risks for SCA and create standardized protocols.

## 1. Introduction

Sudden deaths of young athletes have a significant impact on those around them, and sudden deaths among student athletes are of great social concern. Reports from the United States indicate a yearly occurrence rate of sudden death ranging from 50 to 100 per 1000 deaths [1], with athletes facing 2–4 times higher risk than the general population [2]. Although sudden cardiac death (SCD) generally correlates more with age, sports-related sudden death predominantly affects the young, with approximately 40% of cases occurring in patients under 18 years [3]. Coronary artery disease, hypertrophic cardiomyopathy (HCM), arrhythmogenic right ventricular cardiomyopathy (ARVC), and coronary artery anomalies are notable causative factors of sudden death in young athletes. HCM accounts for 10%–51% of such cases, whereas ARVC contributes to 10% [4–8]. Coronary artery anomalies, which are prevalent in 1.3% of the general population [9], are detected in 24% of athletes who succumb to SCD [8].

Although the implementation of social safety measures—such as the availability of automated external defibrillators at sports venues—has advanced the prevention of sports-related sudden death, not all cases of sudden cardiac arrest (SCA) can be prevented. To efficiently prevent SCA, which occurs at a very low frequency, it is essential to identify high risk athletes in advance and provide appropriate evaluation and treatment. Effective and efficient screening requires first identifying the risk factors for SCA. Among young individuals, a higher incidence of SCA has been reported particularly among those of university age [7, 10]. Therefore, this study aimed to delineate the distribution, frequency, and risk factors of sudden deaths among Japanese university student athletes, an age group with a notably high incidence of SCA, thereby contributing to the academic discourse on safeguarding athlete welfare.

## 2. Materials and Methods

### 2.1. Study Design

In this cross-sectional observational study, the data were collected through a web-based survey conducted from June 2022 to October 2022. This study was approved by the Ethics Committee of Keio University (approval number: 20211158), was conducted in accordance with the principles of the Declaration of Helsinki [11], and followed the Strengthening the Reporting of Observational Studies in Epidemiology Extension for Sports Injury and Illness Surveillance consensus statement [12]. All participants provided informed consent prior to participation. The questionnaire used in this study was validated using the Delphi method. Eleven individuals (four orthopedic surgeons, one internist, one neurosurgeon, four athletic trainers, and one epidemiologist), along with four external experts, evaluated the validity, feasibility, and clarity of the questionnaire developed based on the International Olympic Committee consensus statement. Each item was rated on a 9-point scale. If all items received a score of 7 or higher, the draft was considered approved and adopted as the final version. If any item received a score of 6 or lower, the questionnaire was revised based on free-text comments, followed by a second round of review. As a result, the average score for all items exceeded 7 in the first round, and the questionnaire was finalized accordingly.

### 2.2. Participant Recruitment

Potential participants were invited from 219 universities and 36 sports organizations associated with the Japanese Collegiate Athletic Association. University sports departments and organizations were invited through letters and phone calls, and the sports managers or athletic trainers were asked to distribute the link to the information sheet of the study and consent forms to the athletes that were interested. After reviewing the study information sheet and providing informed consent, the athletes were invited to participate. All survey data were anonymized, and participation was voluntary. Informed consent was obtained from all participants prior to the administration of the survey.

### 2.3. Survey

A survey was conducted using a custom-built website. The questions were adopted from the recently published Japanese Society of Clinical Sports Medicine and Japanese Society for Athletic Training consensus document [13] and modified to suit the collegiate population.

Questions regarding the athlete characteristics (age, sex, weight, height, and sport) and personal and familial medical history (heart failure, arrhythmia, pacemaker implantation, syncope, and SCA) were asked. SCA and other histories were based on self-reporting. The data were anonymized.

### 2.4. Statistical Analysis

All statistical analyses were performed with SPSS (IBM Corp. Released 2021. IBM SPSS Statistics for Macintosh, Version 28.0. Armonk, NY: IBM Corp). Qualitative and quantitative data were obtained from a survey. Qualitative data were summarized as frequencies, and continuous data were summarized as means and standard deviations. For discrete data, counts and percentages were calculated. Statistical significance was set at *p* < 0.05.

We assessed the associated personal and familial medical histories of patients with SCA using Fisher's exact test for categorical data. For each outcome variable, we estimated the odds ratios (ORs) with 95% confidence intervals (CIs) using a logistic regression approach.

## 3. Results

### 3.1. Participants Characteristics

Of the 24,866 unique clicks on the survey webpage, 11,030 (44.3%) responses were recorded. However, 32 of these were submitted by nonathletes and were therefore excluded. The baseline characteristics of the remaining 10,998 participants were consistent with those reported by Kimura et al. using the same database [14], as shown in Table 1.

The average age of the athletes who responded to the survey was 19.9 (±1.3) years, and of all the respondents, 62.3% were men. Lacrosse players accounted for the highest proportion, with 1689 (15.4%) respondents.

### 3.2. Personal and Family Medical History

The profiles of the six athletes who experienced SCA are shown in Table 2.

The personal and medical histories of the participants are presented in Table 3. Of the 10,998 athletes with background data, 137 individuals who provided incomplete responses to questions related to SCA risk were excluded. Therefore, a total of 10,861 participants were included in the analysis of ORs presented in Table 3.

Among the athletes with SCA, two (33%) had a history of arrhythmia, whereas four (67%) had a history of syncope. Arrhythmia and syncope were significantly more prevalent among athletes with SCA than among those without SCA (*p* < 0.01). Similarly, families of athletes with SCA also exhibited a higher frequency of heart failure, arrhythmia, and syncope (*p* < 0.01).

None of the athletes with SCA had a history of heart failure or cardiac device implantation. There was no significant difference in the prevalence of heart failure or device implantation between the athletes with and without SCA. However, a family history of heart failure was significantly more common among athletes with SCA (*p* < 0.01).

### 3.3. Risk Factors of SCA in the Athlete

The ORs with 95% CIs were estimated using a logistic regression analysis (Table 4).

A personal history of syncope and family history of heart failure emerged as significant risk factors for SCA among collegiate athletes. Athletes with a history of syncope were found to be 42 times more likely to experience SCA, whereas those with a family history of heart failure were 11 times more likely to experience SCA. However, because of the extremely low incidence of SCA, the 95% CI is notably wide (5.99–293.83), and this should be interpreted with caution. Although arrhythmia was more frequent among athletes with SCA, its CI was wide and nonsignificant, indicating a less reliable association.

## 4. Discussion

The findings of the study suggest that a history of syncope and a family history of heart failure in young athletes increased the risk of SCA by 42- and 11-fold, respectively. The known risk factors for sudden death in the general population include cardiovascular risk factors, such as hypertension, diabetes, dyslipidemia, obesity, smoking, family history of sudden death, and history of cardiac or psychiatric disease [3, 15, 16]. However, the prevalence of these risk factors is expected to be very low among collegiate athletes. Therefore, other risk factors that differ from those of the general population should be considered. Causes of sudden death other than atherosclerotic cardiovascular disease include coronary artery anomalies, nonischemic heart disease, and arrhythmic disorders. In particular, cardiogenic syncope is considered a significant risk factor [17, 18], whereas noncardiogenic syncope is widely acknowledged to have a favorable prognosis. However, the diagnostic process for syncope can be time-consuming and often yields ambiguous results. The present study suggests that syncope—regardless of its etiology (cardiac or noncardiac)—is a significant risk factor for SCA. This finding indicates that, in the field of sports, screening for syncope can serve as a useful criterion for identifying individuals at risk of SCA. Regarding family history, a family history of sudden death has been suggested as a potential risk factor for SCA [16]. However, no studies have reported an association between a family history of heart failure and increased SCA risk. Given that heart failure is more prevalent than sudden death, a family history of heart failure may serve as a useful marker in screening high-risk athletes.

Males and people of African American descent have previously been reported as risk factors for SCA [19–21]; however, no sex-related differences were observed in the present study. Past studies have reported that SCA is more common in basketball, soccer ball, and running [7, 22, 23]. A higher intensity and longer duration of exercise have also been reported as risk factors [24–26]. Although the present study did not find significant differences in any specific sport, judo, volleyball, and softball, and ultimate all appeared to be sports with relatively high exercise intensity, which is consistent with previously reported trends. Furthermore, it has been suggested that the high participation rate of males in these sports may have influenced sex differences in the prevalence of SCA among males [7], which may partly explain why no sex-related differences were observed in the present study.

There is currently no unified view on the prescreening of athletes, and recommendations differ between countries and organizations. American Heart Association recommends screening by interview and physical examination of the athlete [27], whereas European Society of Cardiology recommends routine electrocardiography as a screening tool [28]. Although routine electrocardiography has shown promise in increasing diagnostic rates, concerns about its cost-effectiveness persist. Given that syncope was identified as a major risk factor for SCA, taking a detailed history of syncope could be a useful first step in identifying high-risk individuals. Such cases may benefit from further evaluation with electrocardiography and echocardiography. A current challenge in predicting SCD lies in discerning meaningful electrocardiographic changes. Sheikh et al. performed genetic testing on athletes with T-wave inversions on electrocardiography and identified genetic abnormalities associated with several cardiomyopathies and ion channel diseases [29]. In contrast, Cronin et al. reported that genetic testing in athletes with T-wave inversions revealed only variants of uncertain significance [30]. Efforts have been made to apply artificial intelligence to electrocardiographic interpretation for SCA prediction [31], and such technologies may improve risk stratification in athletes.

In approximately 30% of SCD cases in young individuals, the cause remains unidentified even after autopsy [32]. Mellor et al. reported that genetic mutations were found in 17% of these unexplained cases [25] and that a history of syncope is independently associated with genetic abnormalities [33]. Some genetic variants have been significantly associated with SCD [34–36]. Although routine genetic testing for all individuals is impractical, it may be useful in high-risk cases. Identifying high-risk populations through primary screening can enhance the predictive value of electrocardiography, echocardiography, and genetic testing for SCA. Collecting self-reported histories of syncope and family history of heart failure may provide a simple and practical approach for initial screening.

The limitations of the current study include the possibility of recall bias and cognitive bias owing to the retrospective nature of the survey, which may affect the accuracy of the reported data, such as severity. The web-based nature of the survey may also limit the generalizability because of voluntary response bias. Selection bias in this study cannot be ignored. It is assumed that athletes who accessed the website and completed the questionnaire tend to be more conscientious individuals within the overall population, which may have introduced a certain degree of bias. Since the respondents were all individuals who were actively participating in sports at the time of the survey, those who had withdrawn because of injury or other reasons were not included. Therefore, selection bias and sampling bias cannot be ruled out. Since the definition of SCA was based on self-report, there is a risk of misclassification bias. Moreover, the underlying causes of the reported SCA events remain unclear. Although this study administered the questionnaire to patients who recovered from SCA, athletes who died from SCA were not included in the study. As SCA was a rare outcome in our study (*n* = 6), there are limitations in the application of the logistic regression model. This results in wide CIs and high uncertainty. Because only Japanese college student athletes were included in the study, the results may not be generalizable to other regions and races.

## 5. Conclusions

Sudden deaths among athletes remain a problem that needs to be resolved. This study found that a history of syncope and family history of heart failure significantly increased the risk of SCA in Japanese college athletes. Although a thorough prescreening examination of athletes is generally not recommended, close examination of high-risk cases is worth considering. Further research is needed to stratify the risks for the disease and create standardized protocols.

## Figures and Tables

**Table 1:** Characteristics of Japanese collegiate athletes.

Characteristic	All (*n* = 10,998)	Males (*n* = 6848)	Females (*n* = 4094)	Sex unspecified (*n* = 56)
	100%	62.3%	37.2%	0.5%
Age (years), mean ± SD	19.9 ± 1.3	19.9 ± 1.4	19.8 ± 1.3	20.1 ± 1.4
Height (cm), mean ± SD	168.3 ± 8.6	173.1 ± 6	160.2 ± 5.8	165.7 ± 10.0
Weight (kg), mean ± SD	66.2 ± 14.1	72.7 ± 13.3	55.5 ± 7.1	61.4 ± 13.7
BMI, mean ± SD	23.2 ± 3.6	21.6 ± 2.3	21.6 ± 2.3	22.3 ± 3.9
Sporting experience (years), mean ± SD	8.0 ± 4.9	8.1 ± 5.0	7.9 ± 4.8	7.7 ± 4.5
Sports, *n* (%)				
Lacrosse	1689 (15.4)	701 (10.2)	978 (23.9)	10 (17.9)
Softball	1242 (11.3)	300 (4.4)	993 (22.8)	9 (16.1)
Baseball	1212 (11.0)	1208 (17.6)	1 (0.0)	3 (5.4)
American football	896 (8.1)	887 (13.0)	5 (0.1)	4 (7.1)
Soccer	883 (8.0)	697 (10.2)	183 (4.5)	3 (5.4)
Rugby	829 (7.5)	794 (11.6)	34 (0.8)	1 (0.0)
Judo	564 (5.1)	376 (5.5)	183 (4.5)	5 (8.9)
Track	531 (4.8)	386 (5.6)	143 (3.5)	2 (3.6)
Basketball	433 (3.9)	117 (1.1)	314 (7.7)	2 (3.6)
Volleyball	334 (3.0)	105 (1.5)	225 (5.5)	4 (7.1)
Others (74 sports)	2385 (21.7)	1277 (18.6)	1095 (26.7)	13 (23.2)

Abbreviations: BMI, body mass index; SD, standard deviation.

**Table 2:** Profiles of the six athletes with SCA.

	1	2	3	4	5	6
Sex	Female	Male	Female	Female	Male	Male
Age (year)	22	19	20	19	19	20
Year of study	4	1	1	1	2	3
Height (cm)	176	160	170	154	180	177
Weight (kg)	72	60	60	50	92	65
Sport	Volleyball	Judo	Softball	Softball	Judo	Ultimate
Personal medical history						
Heart failure	−	−	−	−	−	−
Arrhythmia	−	−	+	−	−	+
Device	−	−	−	−	−	-
Syncope	−	+	+	+	−	+
Deafness	−	−	−	−	−	−
Family medical history						
Heart failure	−	−	+	−	+	−
Arrhythmia	−	−	+	−	+	+
Device	−	−	−	−	−	−
Syncope	−	−	+	−	−	+
Deafness	−	−	+	−	−	−
Sudden death	−	−	+	−	−	−

Abbreviation: SCA, sudden cardiac arrest.

**Table 3:** Prevalence of personal and family history.

	(a) Athletes with SCA (*n* = 6)	(b) Athletes without SCA (*n* = 10,855)	Total (*n* = 10,861)	*p* value between (a) and (b)
Mean age (years)	20 ± 1	20 ± 1	20 ± 1	0.95
Year in school	2 ± 1	2 ± 1	2 ± 1	0.52
Height (cm)	168 ± 9	169 ± 10	168 ± 9	0.71
Weight (kg)	66 ± 14	66 ± 14	66 ± 14	0.95
Sex, *n* (%)				
Male	3 (50)	6726 (62)	6729 (62)	0.53
Female	3 (50)	4074 (38)	4077 (38)	
Personal medical history				
Heart failure, *n* (%)				
Yes	0 (0)	11 (1)	11 (1)	0.94
No	6 (100)	10,844 (99)	10,850 (99)	
Arrhythmia, *n* (%)				
Yes	2 (33)	314 (3)	316 (3)	< 0.01
No	4 (67)	10,541 (97)	10,545 (97)	
Devices, *n* (%)				
Yes	0 (0)	5 (1)	5 (1)	0.95
No	6 (100)	10,850 (99)	10,856 (99)	
Syncope, *n* (%)				
Yes	4 (67)	329 (3)	333 (3)	< 0.01
No	2 (33)	10,526 (97)	10,528 (97)	
Family medical history				
Heart failure, *n* (%)				
Yes	2 (33)	239 (2)	241 (2)	< 0.01
No	4 (67)	10,572 (98)	10,576 (98)	
Arrhythmia, *n* (%)				
Yes	3 (50)	492 (4)	497 (4)	< 0.01
No	3 (50)	10,241 (96)	10,244 (96)	
Devices, *n* (%)				
Yes	0 (0)	266 (2)	266 (2)	0.69
No	6 (100)	10,479 (98)	10,485 (98)	
Syncope, *n* (%)				
Yes	2 (33)	101 (1)	103 (1)	< 0.01
No	4 (67)	10,652 (99)	10,656 (99)	

Abbreviation: SCA, sudden cardiac arrest.

**Table 4:** Odds ratio for SCA.

Characteristics	*β* (SE)	Wald	Odds ratio	95% CI	*p* value
*Personal medical history*					
Arrhythmia	0.64 (1.23)	0.28	1.91	0.17–21.34	0.6
Syncope	3.73 (0.99)	14.17	41.98	5.99–293.83	< 0.01

*Family medical history*					
Heart failure	2.37 (1.19)	3.97	10.74	1.03–111.19	0.05
Arrhythmia	1.77 (1.07)	2.72	5.86	0.71–47.93	0.09
Syncope	2.00 (1.15)	3.06	7.43	0.78–71.08	0.08

Abbreviations: CI, confidence interval; SCA, sudden cardiac arrest; SE, standard error.

## Data Availability

The data that support the findings of this study are available within the manuscript and are available from the corresponding author upon reasonable request. The source data are provided in this study.
